# Diagnostic stewardship to limit repeat plasma cytomegalovirus viral load testing

**DOI:** 10.1186/s12879-023-08355-0

**Published:** 2023-06-09

**Authors:** Akeatit Trirattanapikul, Ekawat Pasomsub, Sukanya Siriyotha, Oraluck Pattanaprateep, Angsana Phuphuakrat

**Affiliations:** 1 Division of Infectious Diseases, Department of Medicine, Faculty of Medicine Ramathibodi Hospital, Mahidol University, 270 Rama VI Road, Ratchathewi, Bangkok, 10400 Thailand; 2 Department of Pathology, Faculty of Medicine Ramathibodi Hospital, Mahidol University, Bangkok, Thailand; 3 Department of Clinical Epidemiology and Biostatistics, Faculty of Medicine Ramathibodi Hospital, Mahidol University, Bangkok, Thailand

**Keywords:** Cytomegalovirus, Diagnostic stewardship, Molecular test, Monitoring, Viral load

## Abstract

**Background:**

Frequent serial monitoring of plasma cytomegalovirus (CMV) viral load caused unnecessary budgets for laboratory testing without changes in treatment. We aimed to implement diagnostic stewardship to limit CMV viral load testing at appropriate intervals.

**Methods:**

A quasi-experimental study was performed. To avoid unnecessary plasma CMV viral load testing, the inpatient electronic pop-up reminder was launched in 2021. In cases with plasma CMV viral load testing was ordered in intervals of less than five days, telephone interview and feedback were performed. Pre-post intervention data was compared in terms of clinical and monetary outcomes. The rate of plasma CMV viral load testing performed in intervals of less than five days was compared between 2021 and 2019 using the Poisson regression model.

**Results:**

After the protocol implementation, there was a significant decrease in the rate of plasma CMV viral load test orders in intervals of less than five days from 17.5% to 8.0% [incidence rate ratio 0.40, *p* < 0.001]. There was no statistically significant difference in the incidence of CMV DNAemia and CMV disease (*p* = 0.407 and 0.602, respectively). As a result, the hospital could save the costs of plasma CMV viral load testing per 1,000 patients performed with intervals of less than five days from 2,646,048.11 to 1,360,062.89 Thai Baht.

**Conclusions:**

The diagnostic stewardship program is safe and helpful in reducing unnecessary plasma CMV viral load testing and costs.

**Supplementary Information:**

The online version contains supplementary material available at 10.1186/s12879-023-08355-0.

## Key points

Frequent serial plasma CMV viral load testing leads to unnecessary costs and laboratory workload. Diagnostic stewardship to limit repeat CMV viral load tests can reduce unnecessary testing without increasing the incidence of CMV DNAemia and CMV disease.

**Supplementary Information:**

The online version contains supplementary material available at 10.1186/s12879-023-08355-0.

## Background

Cytomegalovirus (CMV) infection is one of the most common infections in immunocompromised patients, including solid organ transplant (SOT) recipients [1], hematopoietic stem cell transplant (HSCT) recipients [2], patients with autoimmune diseases who require high-dose immunosuppressive agents [3], patients with hematologic malignancy [4], and critically ill patients [5]. In these patients, CMV infection significantly increases mortality [6–8], graft rejection [6], and the flare-up of pre-existing autoimmune diseases.

According to current clinical practice guidelines, the detection of CMV DNA by quantitative nucleic acid amplification, called CMV viral load, is considered a standard method for detecting CMV replication in clinical specimens [9]. CMV viral load is a useful marker to predict CMV DNAemia, CMV disease, progression of CMV disease and resolution of symptoms in SOT recipients [10]. The optimal intervals for plasma CMV viral load monitoring are five to seven days [11]. Currently, five commercial CMV quantitative molecular assays are approved by the U.S. Food and Drug Administration [12]. Two assays [COBAS AmpliPrep/COBAS TaqMan CMV Test (Roche Diagnostics, Pleasanton, CA, USA) and Abbott RealTime CMV assay (Abbott, Des Plaines, IL, USA)] are available at our hospital.

Currently, serial monitoring of plasma CMV viral load at intervals of less than five days and using different assays for plasma CMV viral load monitoring have been observed in our hospital, as there have been no restrictions on test requests. Frequent requests might cause unnecessary workloads for technicians, as well as unnecessary budgets for laboratory testing. Using different assays might result in wide variability in viral load values. The 1^st^ WHO International Standard for Human CMV for nucleic acid amplification was developed to reduce inter-assay variability [13]. Although a previous study showed that plasma CMV viral load had a good concordance between the Abbott RealTime CMV assay and Roche Cobas Amplicor CMV Monitor Test [14], clinically acceptable limits of result harmonization for CMV viral load measurement in plasma among the assays are still not achieved [15]. Another issue that possibly complicates the interpretation of CMV viral load results includes the specimen type used e.g., whole blood or plasma [11]. However, our hospital laboratory only accepts plasma specimens for CMV viral load testing. This reduces the complexity of CMV viral load results interpretation.

In previous studies, diagnostic stewardship can help physicians use diagnostic tests, such as *Clostridioides difficile* and multiplex molecular panels appropriately [16–19]. However, no previous study has performed diagnostic stewardship of plasma CMV viral load testing. In this study, we aimed to implement diagnostic stewardship to limit CMV viral load testing. The primary outcome of the protocol implementation was a decrease in numbers of patients undergoing plasma CMV viral load testing at intervals of less than five days after protocol implementation without increasing incidences of CMV DNAemia, CMV syndrome, and CMV diseases. The secondary outcomes included cost reduction of plasma CMV viral load testing after protocol implementation in hospital perspective.

## Methods

We conducted a single-center quasi-experimental study at Ramathibodi Hospital (a 1,300-bed university hospital in Bangkok, Thailand). The study was divided into three periods. Before protocol implementation, we conducted a retrospective study from January to December 2019, when there were no restrictions on CMV viral load test ordering. We reviewed plasma CMV viral load testing performed in intervals of less than five days or using different assays for serial monitoring. In 2020, we developed a diagnostic stewardship protocol for plasma CMV viral load testing. The diagnostic stewardship protocol development included the insertion of a pop-up reminder of an appropriate interval and test for CMV viral load monitoring in the inpatient computerized provider order entry (CPOE), the notification system to the investigator, and feedback delivering. After the protocol implementation, we conducted a prospective cohort study between January and December 2021. Data were collected from patients aged more than 18 years old who were admitted and required repeat plasma CMV viral load testing. Since there were some patients aged more than 18 years old under the care of the pediatric department, these patients were excluded from the study. The patients who required plasma CMV viral load testing only once during the study period were not included. CMV DNAemia is defined as the detection of CMV DNA in plasma above a lower limit of detection of the assay [20]. CMV syndrome and disease were defined according to American Society of Transplantation guidelines [21]. The decision to order CMV viral load testing and to start treatment of CMV DNAemia with anti-CMV agent depended on primary or infectious disease physicians’ opinions.

### Interventions

In 2020, we developed a protocol for plasma CMV viral load monitoring stewardship, which involves the frequency of testing and a commercial assay ordered for each patient. The protocol for CMV viral load monitoring was provided to physicians who might have been concerned before the protocol implementation. After protocol implementation, when a physician ordered CMV viral load testing via the inpatient CPOE, there would be a pop-up reminder displaying the information about CMV viral load testing protocol in the Thai language, which is translated as “CMV Viral Load by Cobas Taqman (EDTA-blood); please review the previous CMV viral load assay before ordering (should be the same assay as the previous order). This test should be performed at intervals of approximately one week. If you need to order the test in intervals of less than one week, please consult an infectious disease physician”. When plasma CMV viral load testing was ordered in intervals of less than five days or using different assays for serial monitoring, the information about plasma CMV viral load testing and ordering physicians were sent from the Virology Laboratory to the investigator (A.T.) every morning. The investigator then interviewed ordering physicians about the reason for plasma CMV viral load testing and gave feedback on proper plasma CMV viral load testing to improve ordering in the future. The request was considered intentional or unintentional when the investigator interviewed about the reason for CMV viral load monitoring in intervals of less than five days with the physician. The request was considered intentional if CMV viral load monitoring in intervals of less than five days was sent for specific reasons by the physician. The request was considered unintentional if CMV viral load monitoring in intervals of less than five days was ordered by physicians unaware of the existing previous CMV viral load testing order in intervals of less than five days. The ordering physician could confirm the order for a specific reason. If the ordering physicians decided to cancel the plasma CMV viral load order after the interview, the order was canceled by the investigator. Informed consent was obtained from the physicians interviewed.

### Statistical analysis

The median and interquartile range (IQR) were used for the descriptive statistics. For categorical variables, we used the χ2 or Fisher’s exact test for comparisons between groups. For the comparison of continuous variables, we used a t-test for analysis. Subgroup analyses were performed according to the type of hospital unit (general ward or intensive care unit) and comorbidities. Patients whose tests were ordered at more than one hospital unit were categorized in the unit during their worst condition.

The Poisson regression model was used for the primary outcome analysis. The analysis compared pre- and post-intervention data. If a plasma CMV viral load was requested in intervals of less than five days after another CMV viral load test, the number of test requests was counted. The incidence of plasma CMV viral load testing requested in intervals of less than five days was calculated from the number of tests requested in intervals of less than five days after another CMV viral load test divided by the total number of the test requests. The reasons for plasma CMV viral load ordering in intervals of less than five days and the use of different assays for monitoring before the feedback were collected.

The total costs of plasma CMV viral load testing, including plasma CMV viral load testing in intervals of less than five days, anti-CMV drugs, and procedures related to plasma CMV viral load testing were retrieved from each patient’s invoice and adjusted value to 2021. The costs that occurred in the calendar years 2019 and 2021 were collected. During the study period, the costs of plasma CMV VL testing by COBAS AmpliPrep/COBAS TaqMan CMV Test, and by Abbott RealTime CMV assay were 2,500 and 2,400 Thai Baht, respectively. (One United States Dollar equals 35.0 Thai Baht on December 1, 2022). Anti-CMV drugs collected in this study included ganciclovir, valganciclovir, cidofovir, foscarnet and intravenous immunoglobulin (IVIG). Statistical significance was set at a *P* < 0.05. STATA version 17 (StataCorp, College Station, TX, USA) was used for statistical analyses.

## Results

Baseline characteristics of 609 patients who required repeat plasma CMV viral load testing are shown in Table 1. In 2019, there were 1,764 plasma CMV viral load tests performed in 291 patients. The median (IQR) number of plasma CMV viral load tests was 4 (2–9) times. After protocol implementation, there were 1,674 plasma CMV viral load tests performed in 318 patients in 2021. The median (IQR) number of plasma CMV viral load tests was 3 (2–6) times.

**Table 1:** Baseline characteristics of patients requiring repeat plasma CMV viral load testing

	Pre-intervention (*N* = 291)	Post-intervention (*N* = 318)	*p*-value
Age (years) [median (IQR)]	55 (38–68)	60 (47–71)	0.001
Male gender [n (%)]	154 (52.9)	144 (45.3)	0.060
Ward			0.032
General ward [n (%)]	206 (70.8)	199 (62.6)	
Intensive care unit [n (%)]	85 (29.2)	119 (37.4)	
Comorbidities [n (%)]	271 (93.1)	298 (93.7)	0.772
Solid organ transplantation [n (%)]	74 (25.4)	39 (12.3)	< 0.001
Kidney	67 (90.5)	36 (92.3)	
Liver	4 (5.4)	0 (0.0)	
Heart	3 (4.0)	3 (7.7)	
HSCT [n (%)]	21 (7.2)	25 (7.9)	0.763
ASCT	7 (33.3)	3 (12.0)	
MSD	6 (28.6)	11 (44.0)	
MUD	8 (38.1)	9 (36.0)	
MMD	0 (0.0)	2 (8.0)	
Patients with GVHD	4 (19.1)	11 (44.0)	
Autoimmune diseases [n (%)]	62 (21.3)	66 (20.8)	0.868
SLE	29 (46.8)	24 (36.4)	
Systemic vasculitides	6 (9.7)	7 (10.6)	
Rheumatoid arthritis	5 (8.1)	3 (4.5)	
Inflammatory myositis	1 (1.6)	6 (9.1)	
Systemic sclerosis	4 (6.5)	4 (6.1)	
Leukemia [n (%)]	39 (13.4)	32 (10.1)	0.200
Lymphoma [n (%)]	24 (8.3)	28 (8.8)	0.806
Solid malignancy [n (%)]	14 (4.8)	7 (2.2)	0.078
HIV [n (%)]	13 (4.5)	8 (2.5)	0.187
COVID-19 [n (%)]	0 (0.0)	69 (21.7)	< 0.001
Pretransplant CMV IgG in HSCT patients [n (%)]			0.261
Unavailable	16 (76.2)	16 (64.0)	
D + /R +	4 (19.1)	9 (36.0)	
D + /R-	1 (4.8)	0 (0.0)	
Pretransplant CMV IgG status in solid organ transplant patients [n (%)]			0.717
Unavailable	14 (18.9)	4 (10.3)	
D + /R +	55 (74.3)	33 (84.6)	
D + /R-	3 (4.1)	2 (5.1)	
D-/R +	1 (1.4)	0 (0.0)	
D-/R-	1 (1.4)	0 (0.0)	
Immunosuppressive used [n (%)]			
Corticosteroid	160 (55.0)	181 (56.9)	0.631
Prednisolone > 20 mg/day	79 (27.2)	124 (39.0)	0.002
Tacrolimus	54 (18.6)	34 (10.7)	0.006
Cyclosporin	33 (11.3)	27 (8.5)	0.239
Cyclophosphamide	28 (9.6)	21 (6.6)	0.171
Mycophenolic acid	65 (22.3)	50 (15.7)	0.037
mTOR inhibitor	8 (2.8)	2 (5.2)	0.054
Antithymocyte globulin	17 (5.8)	14 (4.4)	0.420

Abbreviations: *ASCT* Autologous stem cell transplant, *COVID-19* Coronavirus disease 2019, *CMV* Cytomegalovirus, *GVHD* Graft versus host disease, *HSCT* Hematopoietic stem cell transplant, *IgG* Immunoglobulin G, *MSD* Match sibling donor, *mTOR* Mammalian target of rapamycin, *MUD* Matched unrelated donor, *MMD* Mismatch donor

The rate of plasma CMV viral load requested in intervals of less than five days was reduced significantly after the implementation of the pop-up reminder in the inpatient CPOE [from 17.5% to 10.3%; incidence rate ratio (IRR) 0.51, 95% confidence interval (CI) 0.43–0.62, *p* < 0.001]. The rate was further reduced after feedback (from 17.5% to 8.0%, IRR 0.40, 95% CI 0.33–0.43, *p* < 0.001). There were no statistically significant differences in the incidence of CMV DNAemia, plasma CMV viral load > 1,000 IU/mL, and CMV disease (*p* = 0.407, 0.556 and 0.602, respectively) (Table 2). In patients with CMV viral load monitoring in intervals of less than five days, there are four (1.3%) tests in four (1.4%) patients in pre-intervention group and four (3.0%) tests in three (0.9%) patients in post-intervention group that yielded clinically significant results (*p* = 0.715). In the pre-intervention group, all four tests could help physicians to start anti-CMV drugs earlier. In the post-intervention group, three tests could help physicians to discontinue anti-CMV drugs and one test could help physicians to start anti-CMV drugs earlier.

**Table 2:** Incidences of plasma CMV viral load testing performed in intervals of less than five days, CMV DNAemia, and CMV diseases

	Pre-intervention (*N* = 1,764)	Post-intervention (*N* = 1,674)	Incidence rate ratio (95% CI)	*p*-value
Plasma CMV VL testing ordered in intervals < 5 days before the feedback [n (%)]	308 (17.5)	173 (10.3)	0.51 (0.43–0.62)	< 0.001
Plasma CMV VL testing ordered in intervals < 5 days after the feedback [n (%)]	N/A	134 (8.0)	0.40 (0.33–0.43)	< 0.001
	Pre-intervention (*N* = 291)	Post-intervention (*N* = 318)	Risk ratio (95% CI)	*p*-value
CMV DNAemia	124 (42.6)	125 (39.3)	0.92 (0.76–1.11)	0.407
Plasma CMV VL > 1,000 IU/mL	86 (29.6)	101 (31.8)	1.07 (0.85–1.37)	0.556
CMV disease	30 (10.3)	37 (11.6)	1.13 (0.72–1.78)	0.602
CMV pneumonitis	13 (4.5)	20 (6.3)		
CMV retinitis	7 (2.4)	3 (0.9)		
CMV gastrointestinal disease	10 (3.4)	11 (3.5)		

*Abbreviations*: *CI* Confidence interval, *CMV* Cytomegalovirus, *IU* International unit, *mL* Milliliter, *N/A* Not applicable, *VL* Viral load

Subgroup analysis revealed significant reductions in plasma CMV viral load testing performed in intervals of less than five days in almost all subgroups (Table 3). Plasma CMV viral load testing performed in intervals of less than five days was not decreased in HSCT patients (IRR 1.11, 95% CI 0.79–1.58, *p* = 0.542). The incidence of CMV DNAemia, plasma CMV viral load > 1,000 IU/mL, and CMV disease did not increase in this group (*p* = 0.883, 0.966, and 0.900 respectively). The details about CMV DNAemia in SOT recipients, HSCT patients, and patients with autoimmune disease are shown in Supplementary Fig. 1.

**Table 3:** Subgroup analysis of patients requiring repeat plasma CMV viral load testing

	CMV VL testing ordered in intervals < 5 days before the feedback [IRR (95% CI)]	CMV VL testing ordered in intervals < 5 days after the feedback [IRR, (95% CI)]	CMV DNAemia [RR, (95% CI)]	CMV VL > 1,000 IU/mL [RR, (95% CI)]	CMV disease [RR, (95% CI)]
General ward	0.57 (0.46–0.70)**	0.48 (0.39–0.60)**	0.96 (0.75–1.23)	1.05 (0.78–1.42)	1.04 (0.59–1.81)
ICU	0.43 (0.28–0.64)**	0.22 (0.13–0.37)**	0.83 (0.62–1.12)	1.08 (0.72–1.62)	1.16 (0.50–2.68)
Kidney transplant	0.36 (0.17–0.77)*	0.27 (0.12–0.64)*	1.17 (0.75–1.84)	1.27 (0.71–2.27)	1.86 (0.76–4.54)
HSCT	1.11 (0.79–1.58)	1.11 (0.79–1.58)	0.95 (0.44–2.01)	0.98 (0.39–2.47)	0.84 (0.56–12.63)
Autoimmune disease	0.22 (0.13–0.37)**	0.05 (0.18–0.13)**	0.87 (0.61–1.27)	0.98 (0.62–1.54)	1.50 (0.52–4.34)
Leukaemia	0.54 (0.26–1.09)	0.39 (0.18–0.86)*	0.60 (0.17–2.24)	0.61 (0.12–3.12)	0.03 (0.00–0.21)**
Solid malignancy	0.15 (0.37–0.65)*	0.09 (0.01–0.66)*	0.89 (0.42–1.88)	1.33 (0.55–3.22)	0.66 (0.84–5.30)

*Abbreviations*: *CI* Confidence interval, *CMV* Cytomegalovirus, *HSCT* Hematopoietic stem cell transplant, *ICU* Intensive care unit, *IRR* Incidence rate ratio, *IU* International unit, *mL* Milliliter, *RR* Risk ratio, *VL* Viral load

^*^p-value < 0.05

^**^p-value < 0.001

In the CPOE, there were 173 requests with intervals of less than five days before the feedback. Of these, 33.5% of the serial testing in intervals of less than five days was due to unintentional requests. Of the intentional requests, 63.5% were for HSCT recipients (Table 4). Plasma CMV viral load testing performed in intervals of less than five days tended to be performed more in recipients with MUD, recipients with GVHD, and recipients using anti-thymocyte globulin (ATG) (Supplementary Table 1).

**Table 4:** Reasons for serial monitoring plasma CMV viral load ordered in intervals of less than five days

	Number of CMV viral load orders in intervals < 5 days (*N* = 173)
Unintentional prescription	58 (33.5%)
Intentional prescription	115 (66.5%)
Determine the difference between laboratory assay	2 (1.7%)
Pre-bronchoscopy evaluation	3 (2.6%)
Request in HSCT recipients	73 (63.5%)
Physicians concern about CMV DNAemia	37 (32.2%)
Lymphoma patients	23 (59.0%)
Critically ill patients	6 (15.4%)
Acute leukemia patients	6 (15.4%)
Patients with autoimmune disease	1 (2.6%)
COVID-19 patients	1 (2.6%)

*Abbreviations*: *COVID-19* Coronavirus disease 2019, *CMV* Cytomegalovirus, *HSCT* Hematopoietic stem cell transplant

After the emergence of coronavirus disease 2019 (COVID-19), there were 192 plasma CMV viral load requests in 69 moderate-to-severe COVID-19 patients. Of these, 17 (8.9%) and six (3.1%) tests were requested in intervals of less than five days before and after the feedback, respectively. The reason for 95.7% of the serial monitoring plasma CMV viral load testing in intervals of less than five days was due to unintentional requests. The details about characteristics of COVID-19 patients were provided in Supplementary Table 2.

Plasma CMV viral load monitoring using different assays after protocol implementation and feedback was reduced but not statistically significant [38 tests (2.2%) in 32 patients in 2019 and 28 tests (1.7%) in 26 patients in 2021; IRR, 0.67; 95% CI 0.414–1.110, *p* = 0.114]. There were four (0.2%) tests that physicians decided to change from the Abbott RealTime CMV assay to the COBAS AmpliPrep/COBAS TaqMan CMV Test to monitor plasma CMV viral load. There was one (0.06%) test that the author could not contact the physician. There was one (0.06%) test that the physician decided not to change the assay because he/she needed to monitor CMV viral load for the last time. There were two tests (0.1%) in one patient who had intentional laboratory requests to determine the difference between laboratory assays and decide whether to discontinue ganciclovir. The patient was a 58-year-old woman with acquired immunodeficiency syndrome was diagnosed with CMV radiculomyelitis and DNAemia. She had CMV DNAemia when quantified by the Abbott RealTime CMV assay, but not with the COBAS AmpliPrep/COBAS TaqMan CMV Test. However, there were 16 tests (1.0%) in 15 patients that slipped through the diagnostic stewardship process.

The costs per 1,000 patients of total plasma CMV viral load and plasma CMV viral load performed in intervals of less than five days, and anti-CMV drugs were reduced after protocol implementation (15,133,333.33 to 12,804,088.05 Thai Baht, 2,646,048.11 to 1,360,062.89 Thai Baht, and 37,807,832.65 to 19,856,072.01 Thai Baht, respectively) (Table 5). There were 77 patients in the pre-intervention group and 71 patients in the post-intervention group who received anti-CMV drugs (Supplementary Table 3). IVIG was administered for CMV treatment only in the pre-intervention group. There were two (2.6%) patients in the pre-intervention group and four (5.6%) patients in the post-intervention group whom anti-CMV drugs were prescribed beyond the study period (*p* = 0.430).

**Table 5:** Costs per 1,000 patients of plasma CMV viral load testing, anti-CMV drugs, bronchoscopy, and gastrointestinal endoscopy

	Pre-intervention (Thai Baht)	Post-intervention (Thai Baht)
Overall plasma CMV VL testing	15,133,333.33	12,804,088.05
Plasma CMV VL testing in intervals of < 5 days	2,646,048.11	1,360,062.89
Anti-CMV drugs	37,807,832.65	19,856,072.01
Bronchoscopy	514,089.35	365,408.81
Gastrointestinal endoscopy	487,608.25	537,069.18

*Abbreviations*: *CMV* Cytomegalovirus, *VL* Viral load

## Discussion

This study evaluated the impact of diagnostic stewardship on plasma CMV viral load testing. We found that plasma CMV viral load testing in intervals of less than five days was significantly reduced after protocol implementation. The incidence of CMV DNAemia, CMV syndrome, and CMV disease was not altered after the protocol implementation. Moreover, the costs of plasma CMV viral load testing in intervals of less than five days and anti-CMV drugs were also reduced significantly. Our findings on the reduction in laboratory testing and costs were similar to those of previous studies on diagnostic stewardship in *C. difficile* testing [18, 19, 22, 23].

However, plasma CMV viral load monitoring in intervals of less than five days was not decreased in the HSCT subgroup. The plasma CMV viral load was requested biweekly for some patients. Current guidelines recommend that plasma CMV viral load monitoring be performed at least weekly for the first 100 days after HSCT [24]. Previous studies have proposed an intensive strategy for biweekly monitoring of plasma CMV viral load in seropositive umbilical cord blood transplant recipients [25, 26]. Another study found that alemtuzumab increased the risk of CMV infection by 4.8 times, which may require more intensive plasma CMV viral load monitoring [27]. However, no patients had umbilical cord blood transplant recipients or used alemtuzumab in our hospital. Previous studies have found that HSCT with a CMV-negative donor/CMV-positive recipient, patients with GVHD, and unrelated or mismatched donor serostatus were considered major risk factors for CMV reactivation, CMV disease, and CMV recurrence [28, 29]. However, low-dose ATG in patients undergoing MUD HSCT did not increase the incidence of CMV reactivation or CMV disease [30]. In HSCT recipients that required plasma CMV viral load monitoring in our hospital, we found plasma CMV viral load monitoring in intervals of less than five days was performed in patients with major risk factors for CMV reactivation, CMV disease, and CMV recurrence; however, the number of these patients was too small to detect statistical significance, especially in HSCT recipients with MUD, GVHD, and ATG use. Our study showed that CMV reactivation and CMV disease did not significantly increase in patients with major risk factors; however, the number of events was small. Further studies on CMV monitoring in HSCT recipients and recommendations on the optimal frequencies of CMV viral load monitoring in subgroups of HSCT recipients are needed.

Although there was no clinical practice guidelines recommendation for CMV viral load monitoring other than SOT and HSCT recipients, there was frequent CMV viral load monitoring in autoimmune patients. CMV infection has been reported in patients with autoimmune diseases such as systemic lupus erythematosus (SLE), especially in Asia [31]. Approximately one-fourth of patients in this study had autoimmune diseases. Previous study in our hospital reported 58% mortality rate in SLE patients with CMV disease during 2005–2012 [32]. CMV infection in critically ill immunocompetent patients was associated with poor outcome. A systematic review that included the studies in which CMV viral load performed at least weekly demonstrated the rate of CMV infection of 32–33% [33]. With increasing reports of CMV infection in diverse populations, algorithms for CMV viral load monitoring in these populations are necessary.

During the protocol development in 2020, COVID-19 pandemic emerged. From April 2021, our hospital has provided care for many patients diagnosed with severe COVID-19 pneumonia. This led to differences in baseline characteristics between the pre-intervention and post-intervention groups. CMV DNAemia [34, 35] and CMV diseases, such as disseminated CMV infection [36], CMV myocarditis [37], CMV pneumonitis [38, 39], and CMV proctitis [40] have been reported in COVID-19 patients. A recent study described antiviral stewardship in SOT recipients with COVID-19 and suggested weekly pre-emptive monitoring of plasma CMV viral load [41]. In our study, most of the plasma CMV viral load was ordered in intervals of less than five days before the feedback was unintentionally, probably due to the surge in the number of patients with severe COVID-19, resulting in excessive workload, making the plasma CMV viral load testing review more laborious. This demonstrates the benefits of diagnostic stewardship. Following this study, the algorithms for CMV viral load testing were developed and implemented in our hospital. The algorithms are provided in Supplementary Fig. 2–4.

The costs of total CMV viral load testing and CMV viral load testing in intervals of less than five days are reduced corresponding to the reduction in rate of the test. We also found reduction in costs of anti-CMV drugs after protocol implementation. This might partly be explained by the expensive anti-CMV drugs (valganciclovir and IVIG) were prescribed in a higher number of patients and longer duration in the pre-intervention period. The protocol implementation did not result in increased costs of bronchoscopy and gastrointestinal endoscopy.

The strengths of this study included the study comprised a prospective cohort study evaluated the impact of diagnostic stewardship. We also performed telephone interviews after plasma CMV viral load requests, which provided data on the reason for plasma CMV viral load ordered in intervals of less than five days or using different assays. This study has some limitations. First, this was a single-center study; therefore, the results might limit the external validity to other hospitals. Second, the pre-intervention and post-intervention groups had several significant differences, such as increases in critically ill and COVID-19 patients. The comparison between them could lead to potential bias. Third, the protocol for CMV viral load monitoring provided to physicians who might have been concerned before the protocol implementation might also contribute to reduction in CMV viral load monitoring performed in intervals of less than five days. Fourth, this study did not evaluate deaths or adverse events due to anti-CMV drugs or CMV diseases related to restricted plasma CMV viral load testing. Fifth, anti-CMV drug costs occurred after each calendar year were not collected. Finally, only one investigator reviewed the orders, resulting in incomplete diagnostic stewardship as some (37 tests, 2.2%) unintentional plasma CMV viral load tests were performed.

In conclusion, a diagnostic stewardship program is safe and useful for limiting plasma CMV viral load testing at appropriate intervals and reducing unnecessary cost. Therefore, the program should be maintained. An electronic hard-stop alert as part of the CPOE should be developed and studied.

## Supplementary Information

**Additional file 1:** **Table S1.** Characteristics of plasma CMV viral load monitoring in hematopoietic stem cell transplant recipients after protocol implementation. **Table S2.** Characteristics of 69 moderate-to-severe COVID-19 patients requiring CMV viral load monitoring. **Table S3.** The duration of anti-CMV drugs. **Supplementary Figures 1.** CMV DNAemia in solid organ transplant recipients, hematopoietic stem cell transplant recipients and patients with autoimmune diseases. **Supplementary Figures 2.** Guidance for prevention and screening of CMV infection in solid organ transplant recipients. **Supplementary Figures 3.** Guidance for prevention and screening of CMV infection in hematopoietic stem cell transplant recipients. **Supplementary Figures 4.** Guidance for prevention and screening of CMV infection in patients with autoimmune diseases.

## Data Availability

Data are available from the corresponding author upon reasonable request.

## Abbreviations

ATG: Anti-thymocyte globulin
CI: Confidence interval
CMV: Cytomegalovirus
COVID-19: Coronavirus disease 2019
CPOE: Computerized provider order entry
GVHD: Graft-versus-host disease
HSCT: Hematopoietic stem cell transplant
IgG: Immunoglobulin G
IQR: Interquartile range
IRR: Incidence rate ratio
IVIG: Intravenous immunoglobulin
MMD: HLA-mismatched donor
MSD: HLA-matched sibling donor
MUD: HLA-matched unrelated donor
SLE: Systemic lupus erythematosus
SOT: Solid organ transplant
