# Supplementary material for: Diagnostic stewardship to limit repeat plasma cytomegalovirus viral load testing
Source: BMC Infect Dis. 2023 Jun 9;23:387. doi: 10.1186/s12879-023-08355-0 (PMC10251324; doi:10.1186/s12879-023-08355-0)

**Supplementary material**

**Supplementary Tables**

**Table S1.** Characteristics of plasma CMV viral load monitoring in hematopoietic stem cell transplant recipients after protocol implementation

**Table S2.** Characteristics of 69 moderate-to-severe COVID-19 patients requiring CMV viral load monitoring

**Table S3.** The duration of anti-CMV drugs

**Supplementary Figures**

**Supplementary Figures 1** CMV DNAemia in solid organ transplant recipients, hematopoietic stem cell transplant recipients and autoimmune disease.

**Supplementary Figures 2** Guidance for prevention and screening of CMV infection in solid organ transplant recipients.

**Supplementary Figures 3** Guidance for prevention and screening of CMV infection in hematopoietic stem cell transplant recipients.

**Supplementary Figures 4** Guidance for prevention and screening of CMV infection in patients with autoimmune diseases.

**Table S1.** Characteristics of plasma CMV viral load monitoring in hematopoietic stem cell transplant recipients after protocol implementation

|  | CMV VL testing performed in intervals >5 days  [n (%)] | CMV VL testing performed in intervals <5 days  [n (%)] | p-value |
| --- | --- | --- | --- |
| ASCT | 1 (12.5) | 2 (11.8) | 1.000 |
| MSD | 5 (62.5) | 6 (35.3) | 0.389 |
| MUD | 1 (12.5) | 8 (47.1) | 0.182 |
| MMD | 1 (12.5) | 1 (5.9) | 1.000 |
| Patients with GVHD | 3 (37.5) | 8 (47.1) | 1.000 |
| Using ATG | 2 (25.0) | 8 (47.1)^a^ | 0.402 |

Abbreviations: ASCT, autologous stem cell transplant; ATG, antithymocyte globulin; CMV, cytomegalovirus; GVHD, graft versus host disease; MSD, matched sibling donor; mTOR, mammalian target of rapamycin; MUD, matched unrelated donor; MMD, mismatched donor; VL, viral load.

^a^All with HLA-matched unrelated donor

**Table S2.** Characteristics of 69 moderate-to-severe COVID-19 patients requiring CMV viral load monitoring

|  |  |  | N (%) |
| --- | --- | --- | --- |
| Treatment received | | |  |
|  | Intravenous methylprednisolone or high-dose dexamethasone | | 59 (85.5%) |
|  | Tocilizumab | | 9 (13.0%) |
|  | Tofacitinib | | 9 (13.0%) |
|  | Baricitinib | | 11 (15.9%) |
| Outcomes | | |  |
|  | CMV DNAemia | | 26 (37.7%) |
|  | CMV disease | | 6 (8.7%) |
|  |  | Probable CMV pneumonitis | 4 (5.8%) |
|  |  | Probable CMV GI disease | 2 (2.9%) |

Abbreviations: CMV, cytomegalovirus; GI, gastrointestinal

**Table S3.** The duration of anti-CMV drugs

|  | Pre-intervention | Post-intervention | p-value |
| --- | --- | --- | --- |
| Total anti-CMV medications |  |  |  |
| Number of patients | 77 | 71 | 0.273 |
| Duration (days) [median (IQR)] | 24 (14-42) | 24 (14-46) | 0.545 |
| Ganciclovir |  |  |  |
| Number of patients | 74 | 62 | 0.079 |
| Duration (days) [median (IQR)] | 19 (14-30) | 18.5 (14-30) | 0.805 |
| Valganciclovir |  |  |  |
| Number of patients | 23 | 28 | 0.688 |
| Duration (days) [median (IQR)] | 23 (10-58) | 22.5 (12-47) | 0.769 |
| Foscarnet |  |  |  |
| Number of patients | 2 | 1 | 0.608 |
| Duration (days) [median (IQR)] | 5 (3-6) | 9 (9-9) | 0.221 |
| Cidofovir |  |  |  |
| Number of patients | 0 | 2 | 0.500 |
| Duration (days) [median (IQR)] | N/A | 26 (18-33) | .. |
| Intravenous immunoglobulin |  |  |  |
| Number of patients | 6 | 0 | 0.012 |
| Duration (days) [median (IQR)] | 4 (3-5) | N/A | .. |

Abbreviations: IQR, interquartile range; N/A, not applicable.

**Supplementary Figure 1**

**Supplementary Figures 2**


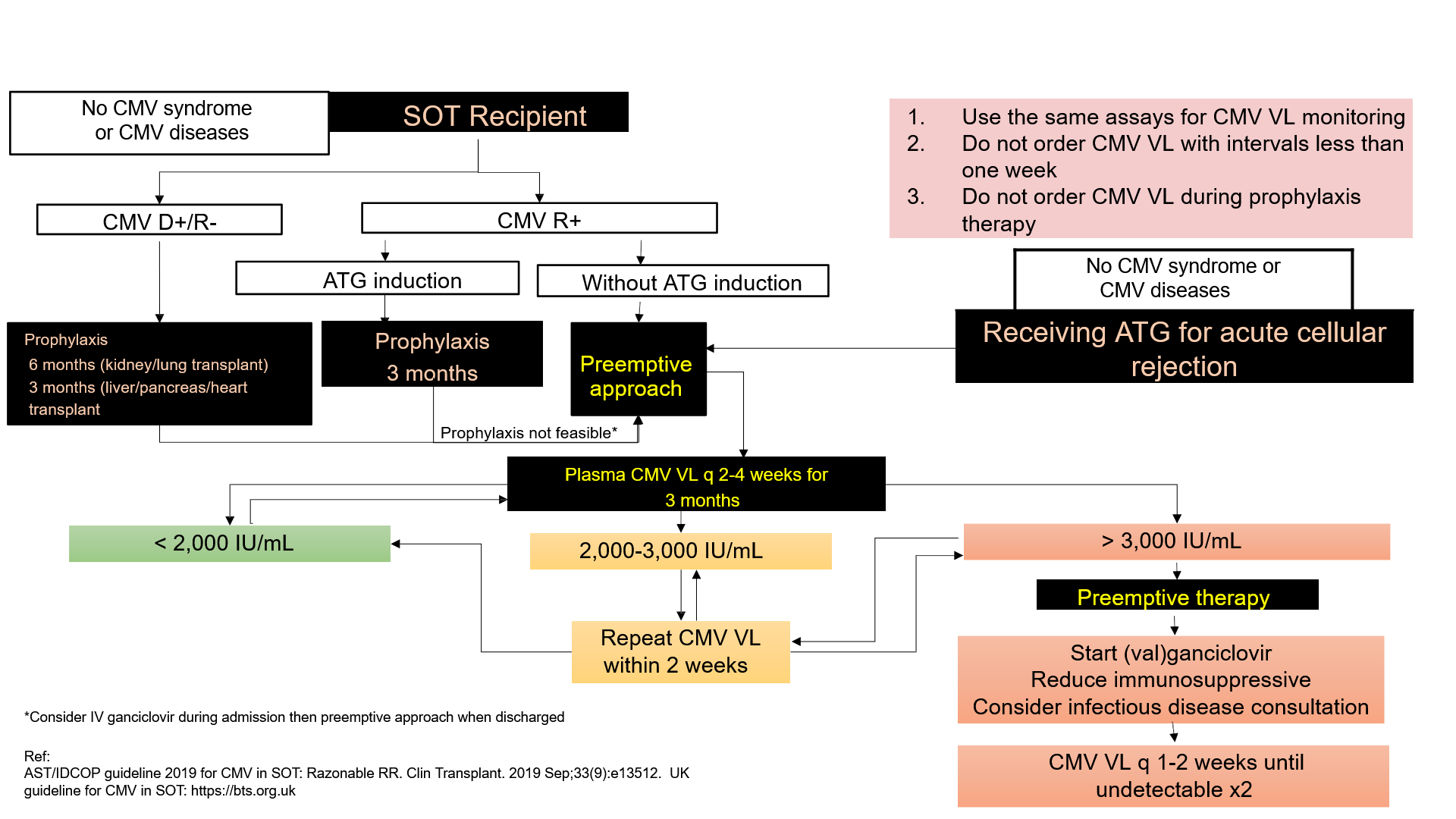


**Supplementary Figures 3**


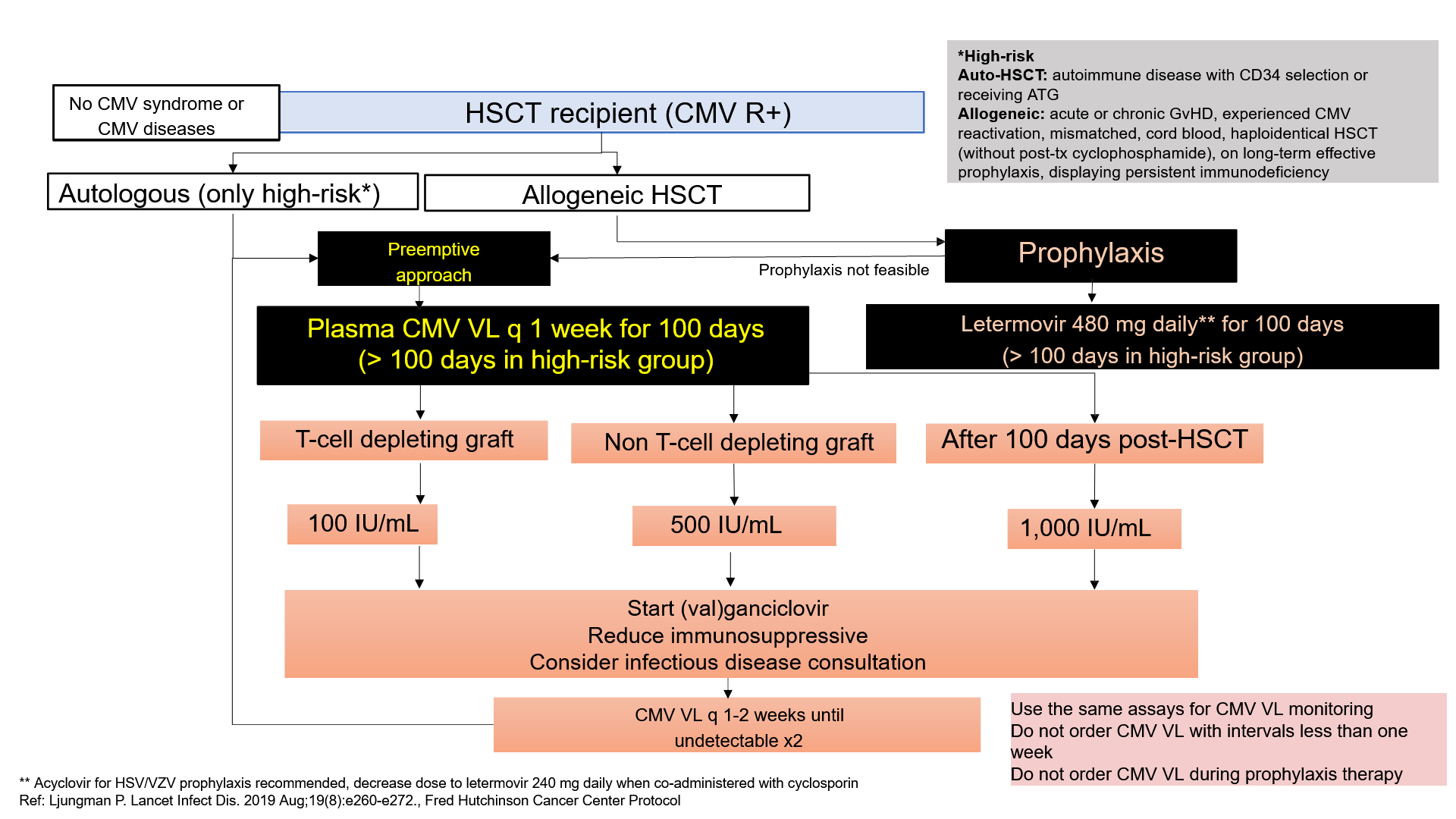


**Supplementary Figures 4**


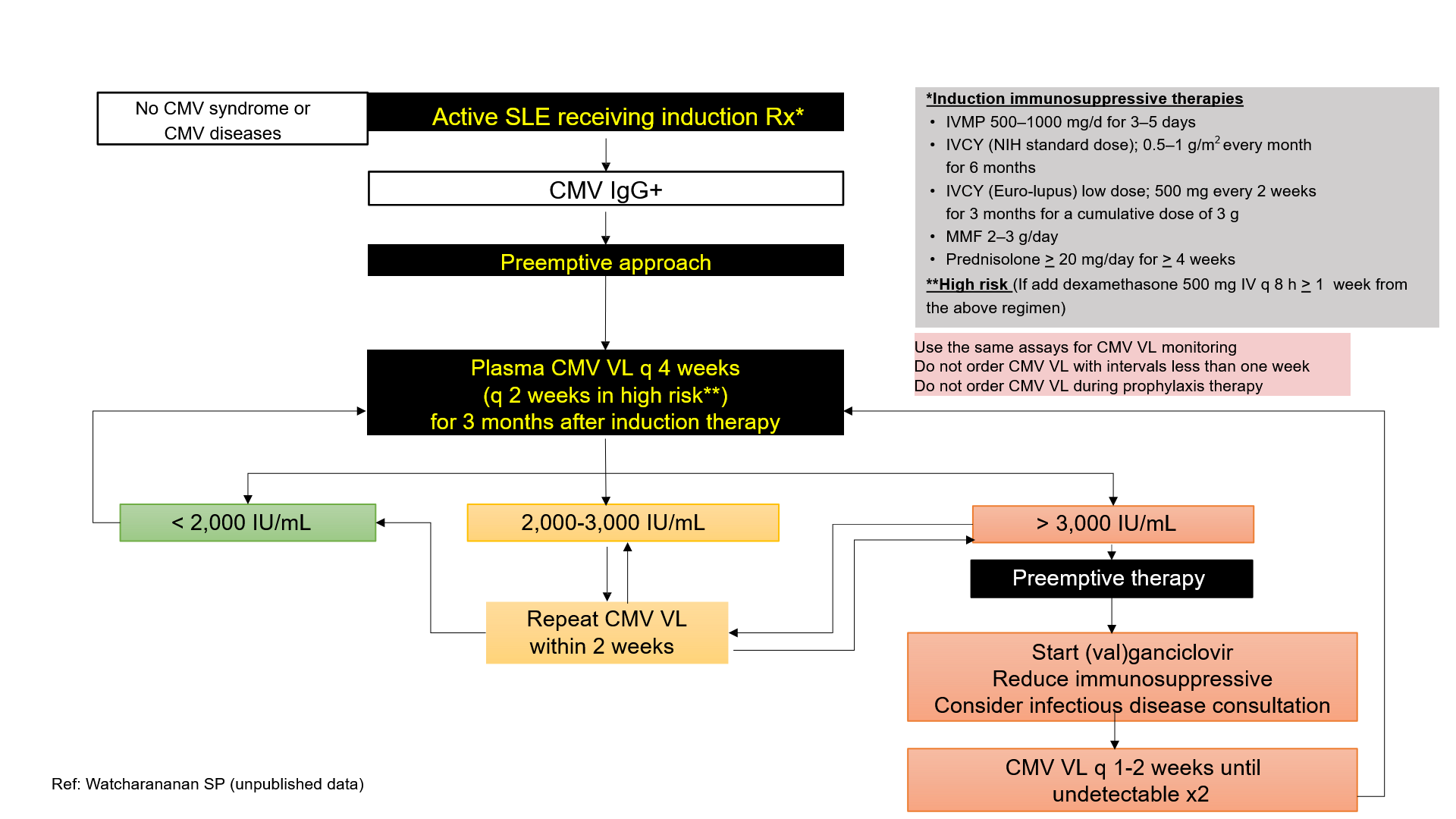

Supplement: Supplementary file 1 — Additional file 1: Table S1. Characteristics of plasma CMV viral load monitoring in hematopoietic stem cell transplant recipients after protocol implementation. Table S2. Characteristics of 69 moderate-to-severe COVID-19 patients requiring CMV viral load monitoring. Table S3. The duration of anti-CMV drugs. Supplementary Figures 1. CMV DNAemia in solid organ transplant recipients, hematopoietic stem cell transplant recipients and patients with autoimmune diseases. Supplementary Figures 2. Guidance for prevention and screening of CMV infection in solid organ transplant recipients. Supplementary Figures 3. Guidance for prevention and screening of CMV infection in hematopoietic stem cell transplant recipients. Supplementary Figures 4. Guidance for prevention and screening of CMV infection in patients with autoimmune diseases. [file 12879_2023_8355_MOESM1_ESM.docx]
